# Supplementary material for: Symptom trajectories of non-cancer patients in the last six months of life: Identifying needs in a population-based home care cohort
Source: PLoS One. 2021 Jun 15;16(6):e0252814. doi: 10.1371/journal.pone.0252814 (PMC8205160; doi:10.1371/journal.pone.0252814)
Supplement: S1 Fig — (DOCX) [file pone.0252814.s001.docx]

**S1 Fig. CONSORT diagram**

DAD=Discharge Abstract Database, RAI= Resident Assessment Instrument for Home Care

Excluded assessments with cancer (*n=30,368*)

Non-Cancer Trajectories (cardiovascular, neurological, respiratory, renal)

*n=33,596 assessments*

Count of repeat assessments (*n=12,823*)

Non-Cancer Demographics (unique individuals)

*n=20,773*

Excluded assessments without 4 disease groups of interest (*n=34,594*)

Unique individuals who died in hospital, DAD dataset 2002-2014: *n= 118,032*

All RAI assessments 2006-2014:

*n=1,412,210*

Merged DAD and RAI

*n= 1,412,210*

Excluded those who do not have a death reported in home or hospital *(n=1,010,418)*

Merged data set, including all RAI assessments, only for those who have died

*n= 401,792*

All assessments within 26 weeks of death

*n= 98,558*

*(86,138 unique individuals)*

Excluded assessments that occurred earlier than 26 weeks prior to death *(n=303,234)*
