# Supplementary material for: Symptom trajectories of non-cancer patients in the last six months of life: Identifying needs in a population-based home care cohort
Source: PLoS One. 2021 Jun 15;16(6):e0252814. doi: 10.1371/journal.pone.0252814 (PMC8205160; doi:10.1371/journal.pone.0252814)
Supplement: S2 Fig — (DOCX) [file pone.0252814.s002.docx]

**S2 Fig: The RECORD statement – checklist of items, extended from the STROBE statement, that should be reported in observational studies using routinely collected health data.**

|  | **Item No.** | **STROBE items** | **Location in manuscript where items are reported** | **RECORD items** | **Location in manuscript where items are reported** |
| --- | --- | --- | --- | --- | --- |
| **Title and abstract** | | | | | |
|  | 1 | (a) Indicate the study’s design with a commonly used term in the title or the abstract (b) Provide in the abstract an informative and balanced summary of what was done and what was found | Done. Title and abstract. | RECORD 1.1: The type of data used should be specified in the title or abstract. When possible, the name of the databases used should be included.  RECORD 1.2: If applicable, the geographic region and timeframe within which the study took place should be reported in the title or abstract.  RECORD 1.3: If linkage between databases was conducted for the study, this should be clearly stated in the title or abstract. | Done. Databases used are named in abstract. Type of data in title.  Done. Region and timeframe in abstract.  Linkage stated in abstract. |
| **Introduction** | | | | | |
| Background rationale | 2 | Explain the scientific background and rationale for the investigation being reported | Done. In introduction. |  |  |
| Objectives | 3 | State specific objectives, including any prespecified  hypotheses | Done. Objective stated in introduction. |  |  |
| **Methods** | | | | | |
| Study Design | 4 | Present key elements of study design early in the paper | Done. In section “study design, participants, and setting” |  |  |
| Setting | 5 | Describe the setting, locations, and relevant dates, including periods of recruitment, exposure,  follow-up, and data collection | Done. In section “study design, participants, and setting” |  |  |
| Participants | 6 | *(a) Cohort study* - Give the | Done. In section “study design, participants, and setting” | RECORD 6.1: The methods of study | Done. Details of inclusion criteria provided in “study design, participants, and setting” section. |

|  |  | eligibility criteria, and the sources and methods of selection of participants. Describe methods of follow-up  *Case-control study* - Give the eligibility criteria, and the sources and methods of case ascertainment and control selection. Give the rationale for the choice of cases and controls *Cross-sectional study* - Give the eligibility criteria, and the sources and methods of selection of participants  *(b) Cohort study* - For matched studies, give matching criteria and number of exposed and unexposed  *Case-control study* - For matched studies, give matching criteria and the number of controls per case |  | population selection (such as codes or algorithms used to identify subjects) should be listed in detail. If this is not possible, an explanation should be provided.  RECORD 6.2: Any validation studies of the codes or algorithms used to select the population should be referenced. If validation was conducted for this study and not published elsewhere, detailed methods and results should be provided.  RECORD 6.3: If the study involved linkage of databases, consider use of a flow diagram or other graphical display to demonstrate the data linkage process, including the number of individuals with linked data at each stage. | Done. References 25-30 include studies validating the relevant aspects of the tool.  Done. This is included as a supplementary Figure 1. |
| --- | --- | --- | --- | --- | --- |
| Variables | 7 | Clearly define all outcomes, exposures, predictors, potential confounders, and effect modifiers. Give diagnostic criteria, if applicable. | Done. This is included in section called “variables” “outcomes” and “covariates” respectively. | RECORD 7.1: A complete list of codes and algorithms used to classify exposures, outcomes, confounders, and effect modifiers should be provided. If these cannot be reported, an  explanation should be provided. | Done. We have included the exact item #s for each variable code, from the assessment tool. Detailed in “variables” “outcomes” and “covariates” respectively. |
| Data sources/ measurement | 8 | For each variable of interest, give sources of data and details of methods of assessment (measurement).  Describe comparability of assessment methods if there is more than one group | Done. Found in “data sources” section. |  |  |
| Bias | 9 | Describe any efforts to address  potential sources of bias | Done. Found in “statistical analysis” section. We conducted a sensitivity analysis to explore potential for selection bias in those who died in hospital vs. home. |  |  |
| Study size | 10 | Explain how the study size was  arrived at | Done. It is a population based cohort is explained in the “study design” section. In first sentence of results, we explain the flow. We include a CONSORT as a supplementary figure 1. |  |  |
| Quantitative variables | 11 | Explain how quantitative variables were handled in the analyses. If applicable, describe which groupings were chosen,  and why | Done. Variables all described in detail. How they are defined (all dichotomous), and appropriate references given for justification of cut offs, where appropriate. |  |  |
| Statistical methods | 12 | 1. Describe all statistical methods, including those used to control for confounding 2. Describe any methods used to examine subgroups and interactions 3. Explain how missing data were addressed 4. *Cohort study* - If applicable, explain how loss to follow-up was addressed   *Case-control study* - If applicable, explain how matching of cases and controls was addressed  *Cross-sectional study* - If applicable, describe analytical methods taking account of sampling strategy   1. Describe any sensitivity analyses | 1. Done. Described in “statistical methods” 2. Done. Subgroups described and defined as 4 disease groups (described in “variables”) 3. Done. Missing data discussed in “discussion.” (no missing data as this is administrative mandatory required data) 4. Done. In “discussion” – no loss to follow up as this is administrative data. 5. Done. Sensitivity analysis described in “statistical analysis” section. |  |  |
| Data access and cleaning methods |  | .. |  | RECORD 12.1: Authors should describe the extent to which the investigators had access to the database population used to create the study population.  RECORD 12.2: Authors should provide information on the data cleaning methods used in the study. | Done. In the title page, we describe who had access to the data and roles of each investigator/coauthor.  Done. In “methods” |
| Linkage |  | .. |  | RECORD 12.3: State whether the study included person-level, institutional-level, or other data linkage across two or more databases. The methods of linkage and methods of linkage quality evaluation should be  provided. | Done. In “data sources” section, we state databases were merged at the individual level. Methods of linkage also stated here. (unique health insurance number). |
| **Results** | | | | | |
| Participants | 13 | 1. Report the numbers of individuals at each stage of the study (*e.g.*, numbers potentially eligible, examined for eligibility, confirmed eligible, included in the study, completing follow-up, and analysed) 2. Give reasons for non- participation at each stage. 3. Consider use of a flow diagram | 1. Done. In supplementary Figure 1 (CONSORT) 2. Elimination criteria and numbers provided in supplementary Figure 1. 3. Done. Flow chart used | RECORD 13.1: Describe in detail the selection of the persons included in the study (*i.e.,* study population selection) including filtering based on data quality, data availability and linkage. The selection of included persons can be described in the text and/or by means of the study flow diagram. | Done. See Supplementary Figure 1 |
| Descriptive data | 14 | 1. Give characteristics of study participants (*e.g.*, demographic, clinical, social) and information on exposures and potential confounders 2. Indicate the number of participants with missing data for each variable of interest 3. *Cohort study* - summarise follow-up time (*e.g.*, average and total amount) | 1. Done. See Results, paragraph 1. See Table 1. 2. N/A as described in discussion 3. N/A as population-based study, all participants had equal time, and we looked at the last 26 weeks of life. In Table 1, we describe the proportion of timing of most recent assessment. |  |  |
| Outcome data | 15 | *Cohort study* - Report numbers  of outcome events or summary measures over time  *Case-control study* - Report numbers in each exposure category, or summary measures of exposure  *Cross-sectional study* - Report numbers of outcome events or summary measures | 1. Done. See Table 1. |  |  |
| Main results | 16 | 1. Give unadjusted estimates and, if applicable, confounder- adjusted estimates and their precision (e.g., 95% confidence interval). Make clear which confounders were adjusted for and why they were included 2. Report category boundaries when continuous variables were categorized 3. If relevant, consider translating estimates of relative risk into absolute risk for a meaningful time period | 1. Done. See Table 2 and results, which include 95% CI. 2. N/A. no continuous variables used. 3. N/A. We report odds ratio. |  |  |
| Other analyses | 17 | Report other analyses done—e.g., analyses of subgroups and interactions, and  sensitivity analyses | Done. Table 2 shows disease groups as rows, and died in hospital (sensitivity variable) as another row. |  |  |
| **Discussion** | | | | | |
| Key results | 18 | Summarise key results with  reference to study objectives | Done. See 1^st^ paragraph of discussion |  |  |
| Limitations | 19 | Discuss limitations of the study, taking into account sources of potential bias or imprecision.  Discuss both direction and magnitude of any potential bias | Done. Included as near last as last two paragraphs of discussion. | RECORD 19.1: Discuss the implications of using data that were not created or collected to answer the specific research question(s). Include discussion of misclassification bias, unmeasured confounding, missing data, and changing eligibility over time, as they pertain to the study being reported. | Done. Last two paragraphs of the discussion address this. |
| Interpretation | 20 | Give a cautious overall interpretation of results considering objectives, limitations, multiplicity of analyses, results from similar  studies, and other relevant evidence | Done. See Conclusion. |  |  |
| Generalisability | 21 | Discuss the generalisability (external validity) of the study  results | Done. See 2^nd^ last paragraph of discussion. |  |  |
| **Other Information** | | | | | |
| Funding | 22 | Give the source of funding and the role of the funders for the present study and, if applicable, for the original study on which  the present article is based | Done. See title page and acknowledgements. |  |  |
| Accessibility of protocol, raw data, and programming  code |  | .. |  | RECORD 22.1: Authors should provide information on how to access any supplemental information such as the study protocol, raw data, or  programming code. | Done. Included in title page. |

*Reference: Benchimol EI, Smeeth L, Guttmann A, Harron K, Moher D, Petersen I, Sørensen HT, von Elm E, Langan SM, the RECORD Working Committee. The REporting of studies Conducted using Observational Routinely-collected health Data (RECORD) Statement. *PLoS Medicine* 2015; in press.

*Checklist is protected under Creative Commons Attribution ([CC BY](http://creativecommons.org/licenses/by/4.0/)) license.
